# The Role of TRPV1 in Type 1 Diabetes

**DOI:** 10.3390/biology14121798

**Published:** 2025-12-18

**Authors:** Kelly Silva-Picazo, Euan R. O. Allan

**Affiliations:** 1 Reno School of Medicine, University of Nevada, Reno, NV 89557, USA; kellysilva@med.unr.edu; 2 Department of Microbiology and Immunology, School of Medicine, University of Nevada, Reno, NV 89557, USA

**Keywords:** autoimmune, diabetes, T1D, TRPV1, transient receptor potential vanilloid

## Abstract

Transient receptor potential vanilloid 1 (TRPV1) is an ion channel expressed in sensory neurons, immune cells, pancreatic islets, and vascular tissues. Initially recognized for its role in thermosensation and nociception, TRPV1 has emerged as a key regulator of immune modulation, β-cell physiology, vascular integrity, and neuroimmune signaling—processes central to the pathogenesis and progression of Type 1 Diabetes (T1D). Experimental evidence demonstrates that TRPV1 exerts opposing effects on β-cell physiology—enhancing insulin release during short-term activation, yet accelerating stress and cell loss under chronic stimulation. In the vascular and renal systems, TRPV1 contributes to hallmark T1D complications, including endothelial dysfunction, nephropathy, and impaired cardiovascular protection, while in the central nervous system it drives neuroinflammation, cognitive decline, and emotional dysregulation. TRPV1 sensitization also accelerates the onset and severity of diabetic neuropathy by amplifying pain and inflammatory signaling pathways. Genetic and epigenetic regulation further links TRPV1 to individual susceptibility and disease progression. Collectively, these findings position TRPV1 as both a disease-modifying factor and a determinant of T1D outcomes, underscoring its potential as a biomarker and therapeutic target in autoimmune diabetes.

## Simple Summary

This review examines how the transient receptor potential vanilloid 1 channel acts as an important regulator of inflammation, metabolism, and nerve signaling in type 1 diabetes. Beyond its well-known role in sensing heat and pain, this channel influences how pancreatic cells produce insulin and how immune cells respond to stress, linking metabolic imbalance to autoimmunity. When its signaling becomes disrupted, it contributes to diabetic complications such as blood vessel dysfunction, nerve pain, and cognitive decline. This review brings together scientific findings that identify the transient receptor potential vanilloid 1 channel as a central connection point between the body’s metabolic, nervous, and immune systems in type 1 diabetes. Understanding and targeting this channel, or the molecular pathways it controls, may lead to new, more precise treatments that protect insulin-producing cells and reduce the long-term complications of autoimmune diabetes.

## 1. Introduction

Transient receptor potential vanilloid 1 (TRPV1) is a non-selective cation channel expressed across a broad range of tissues, including sensory neurons, immune cells, vascular endothelium, and pancreatic islets. Known initially for its role in thermosensation and nociception, TRPV1 has garnered increasing attention in the context of autoimmune diseases like Type 1 Diabetes (T1D). In T1D inflammation, immune dysregulation, oxidative stress, and neuronal dysfunction converge to drive both disease onset and progression. While TRPV1 is part of the broader transient receptor potential (TRP) channel superfamily, which includes transient receptor potential canonical channels (TRPC), transient receptor potential melastatin-2 (TRPM2), transient receptor potential vanilloid-4 (TRPV4), and other members implicated in glucose metabolism and inflammation, several features distinguish TRPV1 within this group [1]. Several TRP channels have been implicated in metabolic regulation, however unlike other TRP channels, TRPV1 brings together metabolic, immune, neuroimmune, sensory, and calcium-dependent β-cell signaling pathways relevant to the pathogenesis of T1D into a single molecular framework [1,2,3,4,5,6]. This convergence positions TRPV1 as a mechanistically and clinically significant channel whose dysregulation may contribute to both the initiation and progression of autoimmune diabetes. For this reason, the present review examines how TRPV1 contributes to multiple facets of T1D pathophysiology.

## 2. TRPV1’s Role in Immune Modulation and Autoimmunity

The TRPV1 receptor is a non-selective cation channel that is highly permeable to calcium ions (Ca^2+^) and activated by various stimuli, including noxious heat (>43 °C), low pH, and capsaicin—the pungent compound in chili peppers [7]. TRPV1 belongs to the larger TRP (transient receptor potential) channel family, which plays critical roles in sensory physiology and cellular signaling. Structurally, TRPV1 is a tetrameric ion channel composed of six transmembrane domains per subunit and is regulated by numerous intracellular signaling pathways, including those involving phosphoinositides, kinases, and second messengers [8,9].

TRPV1 is broadly expressed in both neuronal and non-neuronal tissues. It is most well-known for its presence in primary sensory neurons, particularly nociceptive neurons of the dorsal root and trigeminal ganglia, where it contributes to the detection of painful thermal and chemical stimuli [6,9]. However, TRPV1 is also expressed in pancreatic islet cells, immune cells (including T lymphocytes and macrophages), glial cells, and various components of the central nervous system (CNS), where it influences neuroinflammation and metabolic regulation [10,11,12,13,14].

T1D is a chronic autoimmune disease characterized by the immune-mediated destruction of insulin-producing pancreatic β-cells, leading to lifelong insulin deficiency and hyperglycemia. It is driven by a complex interplay of genetic susceptibility, environmental triggers, and immune dysregulation, particularly involving autoreactive T cells and proinflammatory cytokine signaling [13]. As β-cell mass declines, endogenous insulin production becomes inadequate to maintain normal blood glucose levels, resulting in the clinical onset of diabetes.

Emerging evidence suggests that TRPV1 may play a multifaceted role in T1D pathogenesis, influencing both β-cell function and immune regulation. For instance, TRPV1 activation in sensory neurons has been shown to modulate pancreatic inflammation and islet autoimmunity. Neonatal ablation of TRPV1-expressing sensory neurons in non-obese diabetic (NOD) mice conferred long-term protection from diabetes, implicating a sensory-immune communication pathway in autoimmune β-cell destruction [11]. Additionally, TRPV1 appears to modulate cytokine release and immune cell activation, suggesting it may serve as a regulatory point in inflammation and autoimmunity [7]. This aligns with the findings of other studies that have demonstrated that TRPV channels, including TRPV1, are functionally expressed on T lymphocytes, where they regulate calcium entry, cytokine release, and T-cell activation [8]. Furthermore, TRPV1 is expressed in pancreatic β-cells, where it may influence insulin secretion. Activation of TRPV1 can lead to intracellular Ca^2+^ influx, a key trigger for insulin granule exocytosis [15]. Some studies suggest that TRPV1 may enhance glucose-stimulated insulin secretion under certain conditions, while others report that chronic TRPV1 activation may impair β-cell viability [16,17]. This duality reflects the complex roles of TRPV1 in metabolic tissues.

In summary, TRPV1 is a versatile ion channel with widespread expression and diverse physiological roles, including sensory transduction, neuroinflammation, and immune modulation. Given its prominent expression in sensory neurons, pancreatic islets, and immune cells, TRPV1 is emerging as a potentially important player in the pathogenesis of T1D. This connection is particularly compelling in light of the fact that many autoimmune diseases, including T1D, involve pain and inflammation as clinical features. While opioids and non-steroidal anti-inflammatory drugs (NSAIDs) are commonly used for pain relief, these treatments pose significant risks, including addiction, cardiovascular and gastrointestinal side effects [18]. As a result, there is a growing interest in targeting TRP channels—and TRPV1 specifically—as a safer and more targeted therapeutic strategy.

## 3. Beta-Cell Function and Survival

**Figure 1:**
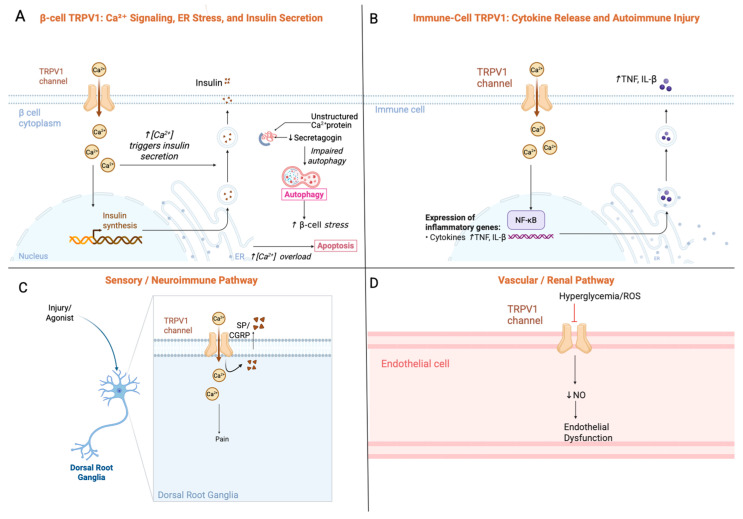
Mechanistic overview of Transient Receptor Potential Vanilloid-1(TRPV1) signaling pathways in Type 1 Diabetes (T1D). (**A**) TRPV1 activation increases Ca^2+^ influx in β-cells, enhancing acute insulin secretion [4,6] but promoting endoplasmic reticulum (ER) stress, C/EBP Homologous Protein (CHOP) activation, and apoptosis under chronic stimulation [5,20,21]. (**B**) In immune cells, TRPV1 mediated Ca^2+^ influx activates NF- κB dependent cytokine pathways (including TNF- α, IL- β) [8,31] and modulates macrophage polarization and high motility group box 1 (HMGB1) signaling [31,32]. (**C**) In sensory neurons, TRPV1 triggers release of substance P (SP) and calcitonin gene-related peptide (CGRP), which enhance dendritic cell activation, T-cell priming, and neurogenic inflammation [6,11,22,23]. (**D**) In endothelial tissues, TRPV1 regulates nitric oxide (NO) production and vascular tone [36,37]; chronic diabetes reduces TRPV1 signaling, contributing to endothelial dysfunction and microvascular injury [38,39,40]. Together, these mechanisms illustrate how TRPV1 influences T1D onset and complications. Arrows indicate direction of effect: ↑ increase; ↓ decrease.

Pancreatic β-cells are essential for glucose homeostasis through insulin production and secretion, however they are highly susceptible to metabolic and inflammatory stress. TRPV1 influences β-cell physiology through direct intracellular mechanisms, including Ca^2+^ entry, glucose stimulated insulin secretion (GSIS), ER stress responses, and apoptosis as well as indirect neuroimmune pathways arising from pancreatic sensory afferents (Figure 1). TRPV1 expression in pancreatic β-cells has been confirmed in rodent models, where it plays a functional role in regulating glucose-stimulated insulin secretion (GSIS) [4]. A TRPV1–secretagogin signaling axis has also been identified, influencing protein turnover and autophagy; chronic TRPV1 activation disrupts this pathway, leading to endoplasmic reticulum (ER) stress and increased β-cell apoptosis [5]. These findings indicate that while TRPV1 activation can acutely enhance insulin secretion, prolonged or dysregulated activity can compromise β-cell survival. Specifically, acute TRPV1 activation promotes a transient Ca^2+^ influx that enhances GSIS via exocytosis. However, chronic or repeated TRPV1 activation produces sustained Ca^2+^ elevation that overwhelms buffering systems and triggers ER stress [19]. This occurs in part through disruption of the secretagogin axis, a Ca^2+^ binding protein essential for vesicle turnover and autophagy regulation [5]. Loss of secretagogin function leads to impaired ER hemostasis, upregulation of C/EBP Homologous Protein (CHOP) and Binding Immunoglobulin Protein (BiP), and activation of pro-apoptotic pathways [20] (Figure 1A). Together, these mechanisms help explain the dual nature of TRPV1 signaling in β-cells, where short-term activation enhances glucose-stimulated insulin secretion, whereas prolonged activation under inflammatory or metabolic stress promotes β-cell dysfunction and apoptosis.

Mechanistic studies have shown that TRPV1 gating is modulated by intracellular protons and calmodulin, which regulate the receptor’s sensitization and desensitization kinetics depending on local pH, calcium concentration, and ligand exposure [21]. Under inflammatory or metabolic stress, TRPV1 activation has been linked to cytotoxic calcium overload and β-cell injury [5,21]. Beyond its expression in pancreatic β-cells, TRPV1 is also localized to sensory afferent fibers innervating the pancreas, where it contributes to neuro-endocrine regulation of islet function. Activation of TRPV1 on these afferents promotes the release of neuropeptides such as substance P (SP) and calcitonin gene-related peptide (CGRP), which in turn enhance insulin secretion through paracrine signaling mechanisms [6] (Figure 1C). Exogenous SP administration has been associated with preserved islet morphology, reduced inflammatory apoptosis, and improved glycemic control in diabetic models [22,23]. Consistent with a net inhibitory afferent tone, ablation or pancreatic denervation of TRPV1^+^ sensory fibers enhances GSIS and improves glucose tolerance without altering β-cell mass or systemic insulin sensitivity; notably these effects were male specific [24].

TRPV1^+^ sensory neurons innervating the pancreas release several neuropeptides that shape islet inflammation [11]. SP and CGRP modulate local cytokine release by macrophages and dendritic cells, enhancing production of IL-1β, TNF-α, and CXCL10 in proinflammatory settings [11,22,23]. These cytokines promote β-cell stress and enhance antigen availability. TRPV1 activation also influences antigen presentation: SP can increase MHC-II expression on macrophages and dendritic cells, while TRPV1-dependent neurogenic inflammation enhances recruitment of CD4^+^ T cells, CD8^+^ cytotoxic T cells, and CCR2^+^ monocytes [11,22]. Through these mechanisms, TRPV1^+^ afferents may modulate the priming and expansion of autoreactive T-cell subsets within the pancreatic microenvironment, thereby linking sensory neuron activity to early autoimmune injury.

Preclinical studies using TRPV1 agonists support its dual role in glycemic control. In streptozotocin (STZ)-induced diabetic rats, capsaicin treatment increased insulin levels, promoted glycogen storage, and lowered blood glucose [25]. Carvacrol, a dietary TRPV1 agonist, has been shown to improve glucose tolerance and preserve islet structure [26]. Insulin and IGF-1 also enhance TRPV1 sensitivity via the PI3K–Akt pathway, amplifying its effects under favorable conditions. However, chronic capsaicin intake has been associated with worsened gut dysbiosis and neuroinflammation in diabetic mice [27]. TRPV1 is highly expressed in a subset of nociceptive sensory neurons, many of which co-express the voltage-gated sodium channel Nav1.8 (*SCN10A*) [28]. Functional studies have demonstrated that Nav1.8^+^ sensory neurons regulate pancreatic insulin output; deletion of insulin receptors in these neurons leads to increased circulating insulin levels, highlighting a neural feedback loop between afferent signaling and β-cell function [29]. Moreover, it has been found that vagal afferents secrete fibroblast growth factor 3 (FGF3), which is essential for glucose-stimulated insulin secretion. Loss of FGF3 disrupts insulin release, consistent with a role for vagal TRPV1-expressing neurons in islet regulation [30]. TRPV1 has also been shown to regulate immune cells. Activation of TRPV1 influences macrophage polarization, shifting populations toward an anti-inflammatory phenotype via Toll-like receptor 4 (TLR4) signaling, with downstream effects on tissue inflammation and immune modulation [31] (Figure 1B). These neuroimmune effects suggest that TRPV1 not only modulates β-cell function directly, but also shapes the local inflammatory environment that impacts β-cell survival.

Finally, TRPV1 is involved in the inflammatory regulation of β-cell survival. Inhibition of HMGB1, a proinflammatory molecule that activates TRPV1, reduces cytokine-induced β-cell injury [32]. Resveratrol has been shown to mitigate TRPV1-related oxidative stress and autophagy in pancreatic tissue via inhibition of the CXCL16/ox-LDL axis [33]. Hovenia dulcis extract has also been found to lower blood glucose and preserve β-cell morphology in diabetic models, potentially through antioxidant effects and TRP channel modulation [34]. Interestingly, *Trpv1* gene deletion in mice has been associated with reduced obesity-induced inflammation and improved insulin sensitivity, highlighting its systemic role in metabolic regulation beyond the pancreas [35]. Similarly, TRPV1 activation in central pathways has been shown to prevent high-fat diet-induced metabolic dysfunction through anti-inflammatory mechanisms [36,37].

## 4. Vascular Dysfunction and Cardiometabolic Complications

While TRPV1 is widely recognized for its role in sensory signaling, growing evidence highlights its essential function in vascular and cardiometabolic regulation—domains highly relevant to the systemic complications seen in T1D. The diabetes-associated lipid mediator 12(S)-HETE activates intracellular TRPV1 in endothelial cells, leading to calcium influx, reactive oxygen species (ROS) generation, and endothelial nitric oxide synthase (eNOS) uncoupling [36]. TRPV1 stimulation by sesamin also promotes eNOS phosphorylation and nitric oxide (NO) production, suggesting potential for restoring vascular function in T1D, where NO bioavailability is diminished [37] (Figure 1D).

TRPV1’s role in neurovascular homeostasis is further supported by evidence that TRPV1-mediated vasodilation—typically triggered by C-fiber afferents—is impaired in T1D mice [38]. Reduced microcirculatory responses were linked to early peripheral neuropathy and decreased CGRP release. Similarly, capsaicin-induced mesenteric artery relaxation is attenuated in T1D rats, correlating with decreased TRPV1 expression and reduced CGRP output [39]. In the kidneys, TRPV1 dysfunction also contributes to T1D-associated end-organ damage. Delayed renal reperfusion following ischemic injury has been linked to altered TRPV1 signaling in diabetic mice, and TRPV1 activity mediates oxidative stress–induced mesangial injury and fibrosis in diabetic nephropathy [40,41,42]. These findings point to TRPV1 as a key mediator of renal microvascular pathology in T1D.

Hypertension is another cardiovascular complication involving TRPV1. TRPV1 activation modulates pressor responses through endothelin-1 signaling, while gastric TRPV1 activation has no effect on rectal motility—highlighting region-specific TRPV1 function and its therapeutic implications in diabetic gastrointestinal dysmotility [43,44]. Cardioprotective effects of TRPV1 are also compromised in T1D. In healthy rats, ischemic postconditioning activates TRPV1 to stimulate CGRP and SP release, protecting the heart from reperfusion injury [43]. This protective mechanism is abolished in T1D rats due to TRPV1 downregulation and neuropeptide depletion, exacerbating cardiac vulnerability.

Altogether, these studies reposition TRPV1 from a nociceptive ion channel to a central regulator of vascular integrity, renal function, and cardiometabolic resilience. Its dysregulation contributes to hallmark complications of T1D, including endothelial dysfunction, impaired perfusion, nephropathy, and cardiac vulnerability—thereby identifying TRPV1 as a promising therapeutic target for restoring neurovascular homeostasis. Although the role of TRPV1 in vascular and renal dysfunction associated with T1D is increasingly acknowledged, much of the mechanistic evidence arises from non-diabetic or acute injury models. A critical gap remains in understanding how chronic hyperglycemia, autoimmunity, and sustained inflammation alter TRPV1 expression and signaling within specific vascular territories. Future investigations employing conditional TRPV1 knockout models in endothelial, renal, and sensory neurons will be essential to delineate these mechanisms and assess their therapeutic potential.

## 5. CNS Dysregulation and Cognitive Impairment

While T1D is traditionally conceptualized as a peripheral autoimmune disease, increasing evidence implicates CNS dysfunction—particularly involving TRP channels such as TRPV1 as a critical mediator of cognitive decline, autonomic dysregulation, and mood disorders in affected individuals [45]. Chronic hyperglycemia and insulin deficiency promote neuroinflammation and oxidative stress, compromising synaptic plasticity and brain network function. Significant dendritic spine loss and spatial memory impairment in T1D rats have been attributed to oxidative and inflammatory mechanisms [46]. These processes likely involve overactivation of redox-sensitive channels such as TRPV1 in hippocampal and cortical neurons. Elevated cytokines and insulin resistance disrupt hippocampal neurogenesis and memory, activating TRPV1-linked calcium dysregulation and neurotoxicity [47,48].

Further evidence of TRPV1’s involvement in T1D cognitive dysfunction showed that antagonizing TRPV1 in the CA1 region of the hippocampus improved spatial learning and reduced anxiety-like behaviors in diabetic rats, suggesting that TRPV1 contributes to hippocampal overexcitation and excitotoxicity [49]. TRPV1 knockout mice exhibit reduced anxiety-like behaviors and impaired hippocampal long-term potentiation, indicating its role in emotional regulation and synaptic plasticity [50]. TRPV1 activation by capsaicin upregulates HDAC2 in the hippocampus, impairing neuronal maturation, synaptic protein expression, and inducing depression-like behavior [8]. Deletion of TRPV1 also increases neural precursor cell proliferation in the dentate gyrus and subventricular zone but reduces differentiation into mature neurons and glia, providing more evidence that TRPV1 is essential for balancing neurogenesis and maturation [51].

The gut–brain–TRPV1 axis also contributes significantly to behavioral regulation and cognitive dysfunction in T1D. Chronic dietary capsaicin exposure—an exogenous TRPV1 agonist—has been shown to exacerbate gut microbiota dysbiosis, increase neuroinflammation, and worsen depressive- and anxiety-like behaviors in NOD mice with T1D [27]. In a study conducted by Zhang et al. (2025) [30], capsaicin-fed diabetic mice exhibited increased gut permeability, elevated serum lipopolysaccharide (LPS) levels, and enhanced microglial activation in the hippocampus and prefrontal cortex—regions critical for memory and emotional regulation. These pathological changes were accompanied by increased expression of pro-inflammatory cytokines (e.g., IL-1β and TNF-α) and stress-related genes, suggesting that TRPV1 overactivation not only disrupts gut microbial homeostasis but also triggers neuroimmune signaling cascades that impair cognitive and affective function. Importantly, these effects were absent in non-diabetic mice, indicating a disease-specific vulnerability wherein TRPV1 serves as a conduit for peripheral inflammatory signals to influence brain function [30]. This supports the notion that aberrant TRPV1 signaling in both enteric and central circuits links gut dysbiosis to CNS complications in T1D, reinforcing its role in mediating mood disorders, cognitive decline, and the broader neuropsychological burden of autoimmune diabetes.

Consistent with this, gut derived inflammatory signals also modulate TRPV1 activity in the CNS through two primary mechanisms. First, endotoxins such as LPS. Activate TRPV1 expressing vagal afferents that project to the nucleus tractus soltarius and parabrachial nucleus, transmitting peripheral inflammatory cues to central circuits [52]. Second, LPS stimulates the release of circulating cytokines, including IL-1β, TNF- α, and IL-6, that access the brain via regions with reduced blood-brain barrier permeability or by activating endothelial TLR4 signaling [53]. These cytokines promote microglial activation and increase central TRPV1 expression and sensitization, thereby linking gut dysbiosis and systemic inflammation to neuroinflammatory responses and behavioral changes observed in diabetes. Together, these pathways illustrate how gut derived immune signals can shape CNS TRPV1 activity through both neural and humoral routes.

TRPV1-evoked neuropeptides, including CGRP and SP, further implicate this channel in neurovascular and emotional regulation. CGRP exerts neuroprotective effects during ischemic brain injury and modulates synaptic signaling in limbic circuits, while SP has been shown to reduce systemic inflammation and preserve β-cell viability in diabetes [22,54]. Both peptides are active within the CNS and may participate in maintaining neurovascular integrity and emotional homeostasis in T1D. Dysregulated TRPV1 activity may therefore alter peptide release, contributing to cognitive dysfunction and mood disturbances through disrupted neuromodulatory signaling.

Finally, elevated markers of inflammation and oxidative stress—including IL-1β and TNF-α—in the hippocampus and prefrontal cortex of diabetic rodents further support TRPV1’s involvement in cognitive and affective dysfunction [55]. These cytokines are well-established TRPV1 sensitizers, and their accumulation suggests that chronic hyperactivation of TRPV1 in central circuits may contribute to mood disturbances and memory impairment in T1D. Neuroimmune mechanisms reinforce this connection: minocycline treatment in diabetic models has been shown to reduce microglial activation and proinflammatory cytokine release, likely through attenuation of TRPV1-mediated signaling [56]. This suggests that pharmacological suppression of microglia–TRPV1 crosstalk may offer neuroprotective benefits, mitigating central sensitization and preserving cognitive function in the context of autoimmune diabetes.

Altogether, this body of work supports the role of TRPV1 as a key integrator of metabolic, inflammatory, and neuroendocrine signals in the brain. In T1D, dysregulated TRPV1 signaling contributes to cognitive decline, anxiety, altered satiety, and central autonomic failure—positioning TRPV1 as both a mechanistic link and therapeutic target in managing CNS manifestations of autoimmune diabetes. While existing studies strongly suggest that TRPV1 is involved in T1D-associated neurocognitive impairment and mood dysfunction, key mechanistic questions remain. Future work should prioritize the use of autoimmune models, conditional TRPV1 knockouts, and circuit-level analyses to definitively map TRPV1’s central role in the gut-brain-immune axis of T1D.

## 6. Sensory Neuropathy and Pain Signaling in Type 1 Diabetes

Diabetic neuropathy is one of the most debilitating complications of T1D, characterized by hyperalgesia, allodynia, and small-fiber dysfunction [45]. Several preclinical mouse models have demonstrated enhanced TRPV1 sensitivity and expression in T1D states. Intracellular signaling pathways including MAPK/ERK activation purinergic receptor signaling, and cytokine mediated sensitization have been implicated in neuropathic pain [57,58,59,60,61,62]. Increased extracellular signal-regulated kinase 1/2 (ERK1/2) phosphorylation in subepidermal nerve fibers has been shown to drive thermal and mechanical hyperalgesia in STZ-induced diabetic rats [57,62]. Since ERK1/2 activation is a known regulator of TRPV1, this provides further evidence regarding the involvement of upstream MAPK signaling in T1D nociceptor hyperexcitability. Similarly, mitochondrial dysfunction and oxidative stress due to defective branched-chain amino acid catabolism in dorsal root ganglia (DRG) neurons have been found to sensitize TRPV1 and thus also contribute to hyperalgesia [63,64].

Purinergic signaling is a major contributor to diabetic neuropathic pain and operates in close coordination with TRPV1 channel activity. The purinergic P2X3 receptors, which are upregulated in DRG neurons in STZ-induced diabetic rats, play a central role in mechanical allodynia by increasing neuronal excitability in response to ATP [32]. These receptors are often co-expressed alongside TRPV1 and share intracellular signaling pathways—including calcium influx and protein kinase activation—that enhance pain signaling under inflammatory and diabetic conditions. In the spinal cord, activation of another purinergic receptor, P2Y13, has been shown to elevate levels of pro-inflammatory cytokines such as IL-1β and IL-6 [57,58,59]. These cytokines are known to sensitize TRPV1 channels, providing an indirect but potent mechanism by which purinergic signaling can amplify TRPV1-mediated nociception. Notably, electroacupuncture (EA) has been shown to reduce diabetic pain by downregulating P2X3 expression and inhibiting its protein kinase C (PKC)-dependent membrane activity [60]. P2X3 is also a key sensitizer of TRPV1, suggesting that EA’s analgesic effects may extend to TRPV1 channel modulation [61]. Further EA studies demonstrated downregulation of P2X3, P2X4, and P2X7 receptors in diabetic DRG neurons [62,63]. These purinergic receptors are upstream regulators of inflammasome activation and cytokine release, which in turn sensitize TRPV1 and TRPA1 channels. The attenuation of mechanical allodynia and thermal hyperalgesia following EA further demonstrates that non-pharmacologic modulation of ion channel expression may be a viable strategy in T1D-associated neuropathy.

Ca^2+^/calmodulin-dependent protein kinase IIα (CaMKIIα) has also emerged as a critical intracellular regulator. Studies have shown that inhibition of phosphorylated CaMKIIα reduced P2X3 expression and relieved neuropathic pain in STZ-induced diabetic rats [63]. Similarly, targeting upstream signaling cascades—such as PKC, cytokine networks, and microglial activation—can influence TRPV1 channel activity and alleviate neuropathic pain [60,64,65]. As shown in one study, administration of neurotrophin-3 (NT-3) reduces thermal hyperalgesia and downregulates TRPV1 in DRG neurons, highlighting the potential for neurotrophic modulation of TRP expression in diabetic pain states [66]. Collectively, these pharmacologic and non-pharmacologic interventions targeting TRPV1 signaling across different models are summarized in Table 1. TRP-mediated pain is not limited to the limbs. In one investigation, diabetic rats with gastropathy exhibited upregulated CCR2 expression in DRG neurons [67]. This chemokine receptor lowers the activation threshold for TRPV1, promoting gastric hyperalgesia and further supporting the role of TRPV1 channels in diabetic visceral pain. Another study introduced a novel corneal sensitivity assay using hyperosmolar eye drops to evaluate small-fiber neuropathy in STZ-induced diabetic rats [68]. The observed reduction in blink and wiping responses reflects hypoactivity or damage to TRPV1-expressing corneal fibers, highlighting the channel’s potential diagnostic and therapeutic relevance in early T1D-associated sensory loss.

While strong preclinical evidence links TRPV1 to diabetic sensory neuropathy, several limitations remain. Most studies utilize STZ-induced diabetes models, which replicate hyperglycemia but not the autoimmune features of Type 1 Diabetes. This limits their ability to capture the full neuroimmune context of TRPV1 dysregulation. Additionally, many mechanistic links—particularly between purinergic receptors and TRPV1—are inferred through correlation rather than direct functional studies. Although non-pharmacologic strategies such as electroacupuncture show promise, their molecular specificity and broader applicability remain uncertain. Further research using autoimmune diabetes models, TRPV1-targeted genetic tools, and tissue-specific expression studies is needed to clarify TRPV1’s role in visceral, cranial, and spinal neuropathies associated with T1D. These efforts will be essential to develop targeted, mechanistically informed treatments for diabetic pain. In summary, TRPV1 acts as a central hub for integrating signals from metabolic stress, neuroinflammation, and calcium imbalance in T1D. Their increased activity contributes to key features of painful diabetic neuropathy, including heightened spinal cord excitability and reduced corneal sensation. Therapeutic strategies targeting these channels—either directly through agents like capsaicin or indirectly via modulation of cytokines, kinases, purinergic signaling, and epigenetic pathways—offer promising avenues for managing T1D sensory complications.

## 7. Genetics, Epigenetics, and TRPV1 Regulation

TRPV1 has emerged as a critical mediator in T1D, with functional roles spanning neuronal, immune, and vascular systems, as well as regulation by genetic and epigenetic mechanisms. Associations between single nucleotide polymorphisms (SNPs) in the *Trpv1* gene and diabetes risk have been reported, indicating that genetic variation may influence channel function, immune reactivity, or vascular tone, thereby modulating individual susceptibility to T1D [69]. For example, the M315I variant of TRPV1 has been associated with an increased risk of T1D in Ashkenazi Jewish populations, suggesting a potential genetic contribution to β-cell autoimmunity or heightened sensitivity to environmental stressors such as oxidative damage or viral exposure [70]. Broader genomic studies also provide additional evidence that TRPV1 expression is influenced by other immune-regulatory genes and microRNAs. For example, *miR-199a* has been shown to downregulate TRPV1 expression in models of inflammatory pain, revealing how post-transcriptional regulation can fine-tune TRPV1’s involvement in immune and nociceptive pathways [71].

Epigenetic regulation of TRPV1 adds another layer of complexity. A long non-coding RNA (*lncRNA*) known as *BC168687* was found to modulate TRPV1 expression in DRG of diabetic rats, and its downregulation significantly alleviated pain symptoms [72]. Histone modifications also play a role: histone acetylation at the TRPV1 promoter region has been shown to increase TRPV1 expression and contribute to thermal hyperalgesia in neuropathic pain models, supporting a mechanism by which inflammatory or metabolic stress epigenetically sensitizes TRPV1-expressing neurons [73]. Additionally, TRPV1 activity is strongly influenced by proinflammatory cytokines and oxidative stress—hallmarks of T1D pathophysiology. Reactive oxygen species (ROS), TNF-α, and IL-1β have been shown to upregulate TRPV1 function or expression, contributing to peripheral sensitization and immune dysregulation [74].

While current studies have begun to uncover genetic and epigenetic mechanisms regulating TRPV1 in the context of diabetes, several limitations remain. Many of the cited investigations focus on neuropathic pain models rather than directly examining TRPV1 activity in pancreatic or immune tissues, which are central to T1D pathogenesis. For example, findings related to microRNA (*miR-199a*), lncRNA *BC168687*, and histone acetylation have not yet been validated in β-cells or immune cells. Additionally, human genetic studies—such as those examining the M315I variant—are limited by ethnic specificity and lack functional validation, leaving it unclear how these polymorphisms alter TRPV1 function or immune behavior in other populations. There is also a lack of longitudinal or stage-specific data examining how TRPV1 regulation changes across the course of T1D development. To address these gaps, future research should investigate TRPV1 regulation using cell-type-specific models, human islet and immune cell systems, and multi-omics approaches in longitudinal T1D cohorts. These studies will be critical to determine whether TRPV1 is a causal mediator or downstream marker in disease progression—and whether it can serve as a personalized therapeutic target in immune and sensory complications of T1D.

It is important to note, however, that most TRPV1 associated genetic and epigenetic findings have been identified in the context of nociception and neuropathic pain rather than in β-cell, immune cell, or pancreatic sensory neuron populations relevant to T1D. For example, the TRPV1 M315I variant, *miR-199a* mediated TRPV1 repression, and the IncRNA BC166687 regulatory network have all been characterized primarily in dorsal root ganglia or pain sensory models, leaving uncertainty about their functional relevance to autoimmune diabetes [70,71]. Whether these molecular modifications alter β-cell stress tolerance, Ca^2+^ signaling, antigen presentation pathways, or immune cell activation remains unknown. Future work should therefore prioritize functional studies in human β-cells, CD4^+^ and CD8^+^ T-cell subsets, macrophages, and pancreatic sensory neurons to determine whether genetic or epigenetic modulation of TRPV1 contributes directly to T1D susceptibility or progression.

## 8. Translational Overview: Human Evidence and Clinical Implications

While much of the mechanistic understanding of TRPV1 in T1D has been derived from murine models, emerging human-based evidence has begun to clarify TRPV1’s relevance in clinical disease. Evidence from studies examining gene expression in human pancreatic tissue have confirmed TRPV1 expression in human pancreatic islets cells, where it participates in calcium signaling pathways that regulate insulin secretion and cellular stress responses [4,5]. Human studies demonstrate that TRPV1 is upregulated in painful diabetic neuropathy, with increased TRPV1-positive nerve fibers in skin biopsies correlating with heat hyperalgesia and clinical pain severity [75]. These findings are complemented by recent work from Tavares-Ferreira et al. (2025), who demonstrated heightened TRPV1 activity and sensitization in human nociceptors from individuals with painful diabetic neuropathy [76]. In addition, TRPV-1 mediated inflammatory pathways contribute to vascular dysfunction in chronic diabetes, particularly through decreased nitric oxide production and altered endothelial responsiveness [77]. Consistent with this, altered TRPV1 signaling in human endothelial and endocrine tissues was identified, linking TRPV1 dysregulation to impaired vasodilatory responses and metabolic stress in patients with diabetes [78].

## 9. Limitations and Future Directions

Despite substantial progress in elucidating the role of TRPV1 in T1D, several limitations persist across current research domains. A major challenge lies in the translational relevance of existing models. Much of what is known about TRPV1’s contribution to β-cell dysfunction, islet inflammation, and autoimmune mechanisms in T1D is derived from murine studies. Although informative, these models do not fully capture human immune responses and TRPV1 expression patterns, which restricts their applicability to human disease processes. To address these gaps, future research should incorporate humanized models, such as in vitro human islet models, mouse models reconstituted with human immune cells, and ex vivo systems in which human islets are co-cultured with immune cells. These approaches would allow more accurate assess TRPV1’s involvement in early β-cell autoimmunity. Additionally, autoimmune susceptible models such as NOD mice engineered to express human TRPV1 or human leukocyte antigen (HLA) haplotypes may provide important opportunities to validate whether TRPV1 modulation alters insulin production, β-cell stress, or disease trajectory.

Additional uncertainties arise from conflicting findings regarding the direct role of TRPV1 in β-cell survival and insulin secretion. TRPV1’s ability to demonstrate both protective but also cytotoxic qualities depending on context, underscores the need for refined models that enable selective manipulation of TRPV1 in β-cells and their afferent sensory circuits. Future studies should also explore how chronic inflammatory environments reshape TRPV1 signaling dynamics in islets throughout the course of disease progression. Longitudinal, disease-stage-specific studies will be essential for determining how TRPV1 activity evolves from preclinical autoimmunity to overt hyperglycemia. In the vascular and renal systems, while TRPV1 is increasingly recognized as a modulator of endothelial function and nephropathy in diabetes, most mechanistic studies are conducted in non-diabetic or acute injury models. The effects of long-term hyperglycemia, autoimmunity, and systemic inflammation on TRPV1 expression across specific vascular beds—particularly in kidney and microcirculatory networks—remain poorly defined. Conditional knockout models targeting TRPV1 in endothelial, renal, and sensory neurons are necessary to elucidate its tissue-specific roles and therapeutic potential in T1D complications.

Another limitation is the narrow focus on TRPV1 as an isolated molecular entity. In reality, TRPV1 functions within a larger network of ion channels that collectively shape inflammatory, metabolic, and nociceptive signaling. TRP channels such as TRPA1, TRPM2, and TRPM8, as well as mechanotransducers such as PIEZO1 and PIEZO2, demonstrate overlapping expression in β-cells, sensory neurons, endothelial cells, and immune lineages [79]. These channels frequently exhibit functional crosstalk, co-activation or shared downstream pathways. Further work should investigate how TRPV1 interacts with these related channels to determine whether its effects in T1D pathophysiology are independent, synergistic, or compensatory. Such studies may uncover multi-channel signaling hubs that could serve as more effective therapeutic targets than TRPV1 alone.

Therapeutic targeting of TRPV1 also presents meaningful challenges that must be acknowledged. First, TRPV1 exhibits strongly context-dependent effects, functioning as a pro-secretory signal during acute activation but driving Ca^2+^ overload, ER stress, and β-cell apoptosis under chronic stimulation [80]. This biphasic behavior means that pharmacologic modulation may have opposite metabolic consequences depending on disease stage, inflammatory environment, or β-cell reserve. Second, systemic TRPV1 agonist or antagonists are associated with significant off-target risks. Clinical trials of TRPV1 antagonists have reported marked hyperthermia, impaired heat sensation, and altered nociception, ultimately limiting their therapeutic window [81,82]. Conversely, widespread TRPV1 activation may induce neurogenic inflammation and autonomic dysregulation [83]. Finally, despite initial enthusiasm, clinical experience with TRPV1 targeting agents in painful diabetic neuropathy has been mixed; several antagonist programs were halted due to thermoregulatory toxicity or inadequate efficacy, and no TRPV1 specific therapy has advanced successfully in diabetes or metabolic disease [84]. Together, these challenges highlight the need for tissue selective, modality specific, or biased TRPV1 modulators supported by improved mechanistic and translated studies.

Finally, the availability of human data from well-characterized patient cohorts remains limited. Technological advances in the mapping of gene activity within intact tissues, combined with improved access to human islets, peripheral nerves, and vascular tissues from individuals with T1D or diabetic complications, will be essential for understanding how TRPV1 functions in specific cell types during disease. Continued expansion of human centered research will be essential to determine TRPV1’s viability as a therapeutic target for the immune, metabolic, vascular, and sensory complications of autoimmune diabetes.

## 10. Conclusions

TRPV1 is a multifunctional ion channel that plays a pivotal role in the pathogenesis and progression of T1D by integrating metabolic, inflammatory, immune, and sensory signals. Across multiple domains, TRPV1 emerges as a key regulator of β-cell function and survival, modulating insulin secretion and cellular stress responses both directly and through neuroimmune pathways. TRPV1 also mediates critical aspects of vascular health and cardiometabolic regulation. Dysregulated TRPV1 signaling contributes to endothelial dysfunction, impaired nitric oxide availability, renal fibrosis, and compromised cardio protection—complications frequently observed in T1D. These vascular effects are compounded by TRPV1’s role in the CNS, where it influences cognitive decline, neuroinflammation, and emotional regulation. The gut–brain–TRPV1 axis further illustrates how peripheral metabolic stressors and microbiome dysbiosis propagate to the brain via neuroimmune signaling, linking T1D to mood and cognitive disorders. In sensory neurons, TRPV1 serves as a major conduit for diabetic neuropathy.

**Figure 2:**
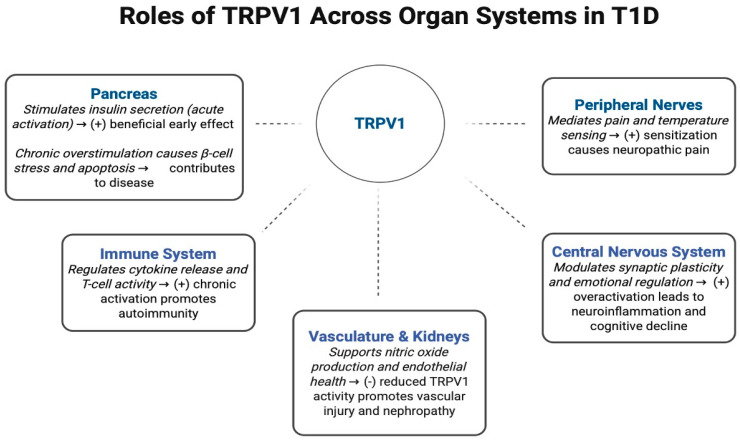
Conceptual model summarizing Transient Receptor Potential Vanilloid-1 (TRPV1) mediated metabolic, immune, vascular, and sensory pathways relevant to Type 1 Diabetes (T1D). This figure integrates findings from published studies demonstrating TRPV-1 dependent effects on β-cell calcium signaling, insulin secretion, ER stress, and apoptosis [4,5,6,19,20,21]; immune modulation through cytokine regulation, macrophage polarization, and neuroimmune signaling [7,11,22,23,31]; sensory neuron sensitization and nociceptive signaling relevant to painful diabetic neuropathy [28,62,63,64]; gut-brain and CNS inflammatory pathways contributing to cognitive and emotional disturbances [27,45,46,47,48,49,52,53,54,55]; and vascular and renal dysfunction mediated by altered TRPV1 signaling [36,37,38,39,40,41,42].

Through interactions with purinergic receptors, cytokines, and intracellular kinases, TRPV1 sensitization promotes hyperalgesia, visceral pain, and small-fiber dysfunction. Interventions such as neurotrophins, electroacupuncture, or cytokine modulation demonstrate that targeting TRPV1-related pathways may offer relief in T1D-associated pain states. Finally, emerging genetic and epigenetic evidence implicates TRPV1 in individual susceptibility to T1D and its complications. SNPs, non-coding RNAs, and histone modifications fine-tune TRPV1 expression in response to inflammation and stress, presenting new opportunities for personalized interventions. Altogether, the literature positions TRPV1 as a dynamic and disease-relevant ion channel with broad regulatory influence across metabolic, immune, vascular, and neural systems in T1D. Figure 2 provides an integrated overview of these system-level interactions, visually summarizing the pathways discussed in this review. Despite promising evidence, substantial gaps remain in model specificity, human translational data, and cell-type-targeted approaches. Addressing these gaps will be critical to determine whether TRPV1 can be effectively leveraged as a diagnostic biomarker or therapeutic target in autoimmune diabetes.

## Figures and Tables

**Table 1:** Therapeutic interventions and modulators of Transient Receptor Potential Vanilloid-1 (TRPV1) signaling in Type 1 Diabetes (T1D) and related metabolic or neuropathic models. These agents include direct TRPV1 agonists and antagonists, indirect pathway modulators, and interventions targeting TRPV1 linked inflammatory, oxidative, or neuroimmune mechanisms.

Compound/ Intervention	Mechanism of Action on TRPV1	Experimental Model	Observed Effect/Outcome	Reference
SP (TRPV1-linked neuropeptide)	Released via TRPV1+ sensory afferents; acts on neuroimmune pathways	STZ-induced diabetic mice	Preserved β-cell morphology; ↓ apoptosis; improved glycemic control	[22,23]
Capsaicin (TRPV1 agonist)	Activates TRPV1; induces Ca^2+^ influx in β-cells; modulates sensory–islet signaling	STZ-induced diabetic rats	↑ Insulin secretion, ↓ blood glucose, improved glycogen storage	[25]
Carvacrol (dietary TRPV1 agonist)	Enhances TRPV1 activity and antioxidant defense	STZ-induced diabetic mice	Improved glucose tolerance, preserved islet morphology	[26]
Vitexin	Inhibits HMGB1 release; attenuates TRPV1-related inflammation	Islet cell culture (LPS-induced pancreatic β-cell injury and apoptosis)	↓ Inflammatory apoptosis, preserved islet cell integrity	[32]
Resveratrol (indirect TRPV1 modulator)	Inhibits CXCL16/ox-LDL pathway; reduces TRPV1-related oxidative stress	STZ-induced diabetic mice	↓ β-cell autophagy, ↓ oxidative injury, improved glucose regulation	[33]
Hovenia dulcis extract	Antioxidant and TRP-modulating effects	STZ-induced diabetic rats	↓ Blood glucose, preserved β-cell structure	[34]
Minocycline (TRPV1–microglia pathway modulator)	Inhibits microglial activation and TRPV1-mediated cytokine release	Streptozotocin (STZ)-induced diabetic neuropathy in mice	↓ Neuroinflammation; improved pain tolerance	[56]
Electroacupuncture (EA)	Downregulates P2X3/P2Y13 receptors that sensitize TRPV1	STZ-induced diabetic rats	↓ TRPV1 sensitization, ↓ hyperalgesia and allodynia	[60,61,62,63]
Neurotrophin-3 (NT-3)	Reduces TRPV1 expression in sensory neurons	Streptozotocin (STZ)-induced diabetic neuropathy in rats	↓ Thermal hyperalgesia; improved sensory function	[66]

Abbreviations: TRPV1, transient receptor potential vanilloid 1; T1D, Type 1 Diabetes; STZ, streptozotocin; SP, substance P; HMGB1, high motility group box 1; EA, electroacupuncture; NT-3, neurotrophin-3; P2X3, purinergic receptor P2X3; P2Y13, purinergic receptor P2Y13; CXCL16/ox-LDL, C-X-C motif chemokine ligand 16/oxidized low density lipoprotein. Arrows indicate direction of effect: ↑ increase; ↓ decrease.

## Data Availability

No new data were created or analyzed in this study. Data sharing is not applicable to this article.

## Abbreviations

The following abbreviations are used in this manuscript:

TRPV1	Transient Receptor Potential Vanilloid 1
CNS	Central Nervous System
T1D	Type 1 Diabetes
NOD	Non-Obese Diabetic
NSAIDs	Non-Steroidal Anti-Inflammatory Drugs
GSIS	Glucose-Stimulated Insulin Secretion
SP	Substance P
CGRP	Calcitonin Gene–Related Peptide
STZ	Streptozotocin
IGF-1	Insulin-Like Growth Factor 1
PI3K	Phosphoinositide 3-Kinase
FGF3	Fibroblast Growth Factor 3
TLR4	Toll-Like Receptor 4
HMGB1	High-Mobility Group Box 1
ROS	Reactive Oxygen Species
eNOS	Endothelial Nitric Oxide Synthase
NO	Nitric Oxide
HDAC2	Histone Deacetylase 2
CA1	Cornu Ammonis 1
LPS	Lipopolysaccharide
IL-1β	Interleukin-1 Beta
IL-6	Interleukin-6
TNF-α	Tumor Necrosis Factor Alpha
TRPA1	Transient Receptor Potential Ankyrin 1
ERK1/2	Extracellular Signal–Regulated Kinase 1/2
MAPK	Mitogen-Activated Protein Kinase
DRG	Dorsal Root Ganglia
P2X3	Purinergic Receptor P2X, Ligand-Gated Ion Channel 3
P2Y13	Purinergic Receptor P2Y13
ATP	Adenosine Triphosphate
PKC	Protein Kinase C
EA	Electroacupuncture
CaMKIIα	Calcium/Calmodulin-Dependent Protein Kinase II Alpha
NT-3	Neurotrophin-3
CCR2	C-C Chemokine Receptor Type 2
SNP	Single Nucleotide Polymorphism
M315I	Methionine-to-Isoleucine Substitution at Position 315
miR-199a	MicroRNA-199a
lncRNA	Long Non-Coding RNA
BC168687	Long Non-Coding RNA BC168687
CXCL16/ox-LDL	C-X-C Motif Chemokine Ligand 16 / Oxidized Low-Density Lipoprotein
LPL	Lysophosphatidylcholine
ER	Endoplasmic Reticulum
TRP	Transient Receptor Potential
CHOP	C/EBP Homologous Protein
BiP	Binding Immunoglobulin Protein
TRPC	Transient receptor potential canonical channels
TRPM2	Transient receptor potential melastatin-2
TRPV4	Transient receptor potential vanilloid-4
HLA	Human leukocyte antigen
